# A Novel Displacement and Tilt Detection Method Using Passive UHF RFID Technology

**DOI:** 10.3390/s18051644

**Published:** 2018-05-21

**Authors:** Xiaozheng Lai, Zhirong Cai, Zeming Xie, Hailong Zhu

**Affiliations:** 1 School of Computer Science & Engineering, South China University of Technology, Guangzhou 510006, China; laixz@scut.edu.cn (X.L.); cscaizhirong@mail.scut.edu.cn (Z.C.); 2 School of Electronic & Information, South China University of Technology, Guangzhou 510641, China; eezmxie@scut.edu.cn; 3 School of Information Engineering, Guangdong University of Technology, Guangzhou 510006, China

**Keywords:** displacement, tilt angle, ultrahigh frequency (UHF), radio frequency identification (RFID), antenna polarization

## Abstract

The displacement and tilt angle of an object are useful information for wireless monitoring applications. In this paper, a low-cost detection method based on passive radio frequency identification (RFID) technology is proposed. This method uses a standard ultrahigh-frequency (UHF) RFID reader to measure the phase variation of the tag response and detect the displacement and tilt angle of RFID tags attached to the targeted object. An accurate displacement result can be detected by the RFID system with a linearly polarized (LP) reader antenna. Based on the displacement results, an accurate tilt angle can also be detected by the RFID system with a circularly polarized (CP) reader antenna, which has been proved to have a linear relationship with the phase parameter of the tag’s backscattered wave. As far as accuracy is concerned, the mean absolute error (MAE) of displacement is less than 2 mm and the MAE of the tilt angle is less than 2.5° for an RFID system with 500 mm working range.

## 1. Introduction

Wireless monitoring has drawn growing interest in the emerging disciplines of Smart City [1] and Internet of Things (IoT) [2]. Automatic detection of displacement and tilt angle is widely deployed in the supply chain industry. In logistics and transportation, some items are expensive and need real-time location monitoring. For example, for an item with a “This side up” marker, the orientation of the item package should be kept [3]. Compared with optical technologies that are used for the detection of visible items, wireless radio signals or electromagnetic signals are more reasonable choices for invisible targets, such as civil infrastructure health monitoring for a Smart City [4].

Recently, distributed wireless sensor networks (WSN) have been widely deployed for wireless monitoring. Applications include a pervasive network of sensor nodes spread over buildings to detect and identify damage in the building structure [5], and thousands of sensors attached to objects to enable real-time estimation of location [6]. A WSN can provide a distributed set of data that is useful to preventing dangerous events in civil infrastructure, or monitoring local behavior of items in supply chain scenarios. However, limited by the cost of system deployment and the requirement of continuous power sources (e.g., a battery), many applications are not feasible with traditional WSN technologies.

**Figure 1:**
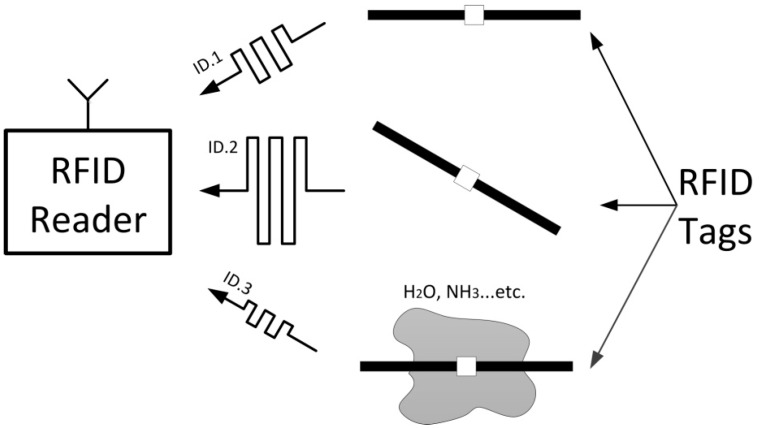
Commercial off-the-shelf (COTS) system of radio frequency identification (RFID) for identification and different sensing applications.

In this paper, a new detection method using radio frequency identification (RFID) technology is proposed to highly reduce the complexity and cost of wireless monitoring systems. RFID is well known as a remote automatic identification technology, which often includes a reader and a tag attached to an object. The tag is energized and interrogated by the reader, through backscattered modulation of the incident continuous wave [7]. An RFID system can provide a radio interface not only for data exchange, but also for sensing capability [8]. Compared with a traditional WSN, an RFID-based sensor has two main cost advantages as follows: Firstly, the battery-less (passive) RFID tag has inherent infinite lifetime, which has a tremendous advantage over high-cost active devices. Secondly, RFID readers and tags are commercial off-the-shelf (COTS) components, instead of a custom-made sensor front-end. As shown in Figure 1, RFID tags achieve different sensing capability by use of uniform electromagnetic information, e.g., drift of resonance frequency or variation of Received Signal Strength Indicator (RSSI) value [9].

Up until now, the published attempts of RFID-based sensors have been focused on physical or chemical factors, such as humidity [10,11,12,13], temperature [14,15,16], gas [16,17,18], strain [19,20], and lab-on-chip (permittivity) [21,22,23]. Based on power measurements [24,25,26], phase detection [27,28,29,30], antenna polarization [31], system analysis [32,33], or multiple tag cooperation [34,35,36], RFID tags can work as sensors for an optional low-cost WSN solution. Different displacement detection approaches can be found in previous works [37,38,39,40,41]. In these papers, tag antennas are physically deformed or damaged by displacement. Unfortunately, as the tag antenna is deformed or broken, it is mismatched, and is hence badly (or even not at all) communicating with the reader. In such a case, it will be very difficult to achieve detection even by using a frequency shift technique, due to the limited allowed RFID bandwidth in USA (902–928 MHz) or Europe (865–868 MHz) [42]. Meanwhile, methods related to tilt detection also have been discussed, which exploits mutual position changing between two tags [43,44,45]. However, accurate angle measurement is difficult to achieve in these attempts.

This paper introduces a novel approach to detecting small displacements and tilt angles of RFID tags. The key idea is to exploit the phase of the backscattered tag response as a carrier of sensing information, while minimizing the degradation of communication performance. Although the phase of the tag response depends on the propagation channel of communication, it can be modified by processing the phase difference of arrival (PDOA) between transmitted and received signals [46]. The methodology of the proposed method is described. Performance testing was carried out with linearly polarized (LP) and circularly polarized (CP) reader antennae. With a 500 mm RFID system working range, the displacement results, which were obtained using an LP reader antenna, demonstrated that the mean absolute error (MAE) was less than 2 mm. Then, based on these displacement results, the tilt angle results, which have a MAE of less than 2.5°, can be obtained using a CP reader antenna with same 500 mm RFID system working range.

The rest of this paper is organized as follows: In Section 2, we present the methodology of the proposed method and the experimental setup. In Section 3, the displacement measurement and tilt angle measurement are described in detail. In Section 4, the limitation conditions of these sensing techniques mentioned above are discussed. Conclusions of the work are drawn in Section 5.

## 2. Materials and Methods

**Figure 2:**
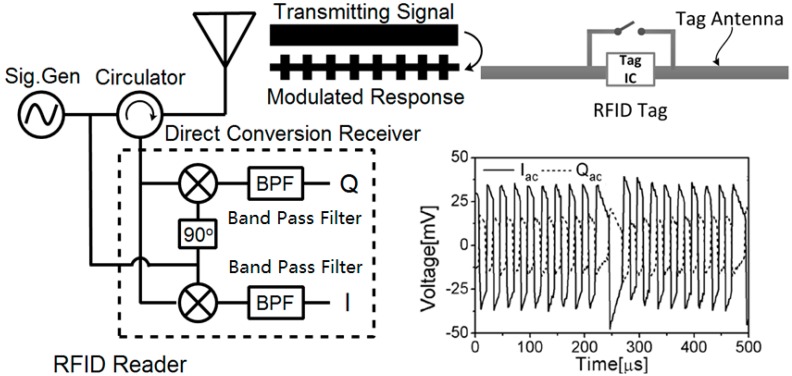
Ultrahigh-frequency (UHF) RFID system block diagram.

As shown in Figure 2, the conventional UHF RFID system consists of a passive tag and an RFID reader, which support fully coherent demodulation of the tag signal. The reader operates in full-duplex mode, and transmits a continuous wave to energize the passive tag. The tag sends a modulated response by alternating its reflection coefficient between two states: State 0 and State 1. State 0 is the matching state between the input impedance of the tag antenna and the tag chip. State 1 is a mismatching state and is caused by shorting the internal circuit of the tag chip. As shown in Figure 2, the tag signal can be divided into an I/Q synchronous sample sequence in the time domain through a phase shifter and a band-pass filter (BPF).

### 2.1. Model

**Figure 3:**
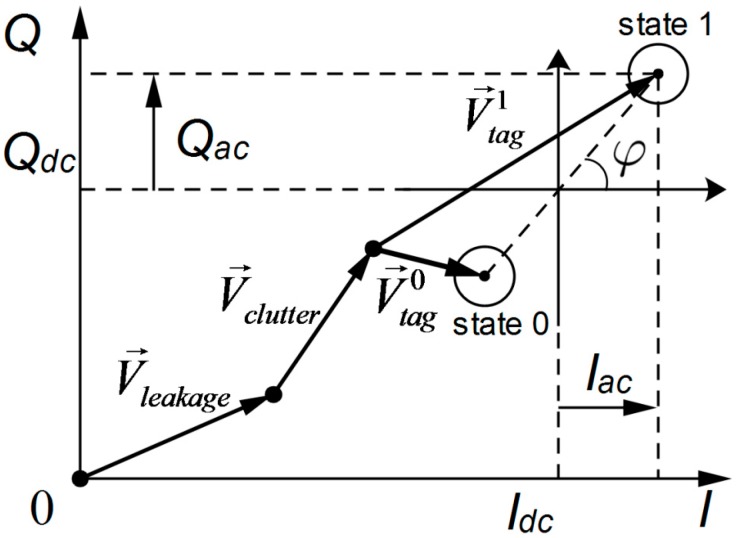
Tag response on the I–Q vector plane [47].

Since the RFID reader can measure both the amplitude and phase of the tag signal by complex demodulation, the tag response can be processed by the reader receiver and transformed into a baseband signal, which can be analyzed on the I–Q vector plane. As shown in Figure 3 [47], it is composed of three components:

(1) $$\vec{V}={\vec{V}}_{leakage}+{\vec{V}}_{scattering}+{\vec{V}}_{tag}.$$

where ${\vec{V}}_{leakage}$ is caused by leakage from the reader transmitter to the receiver; ${\vec{V}}_{scattering}$ is caused by scattering from the surrounding environment; and ${\vec{V}}_{tag}$ is caused by backscattering of the RFID tag, and varied with the states of the tag chip.

Meanwhile, the baseband signal ${\vec{V}}_{}$ can also be represented by the in-phase and quadrature components, denoted as I(t) and Q(t) in the time domain, respectively. Both components are composed of DC and AC parts, which can be shown as follows:

(2) $$I=I_{dc}+I_{ac},\;\;Q=Q_{dc}+Q_{ac}.$$

After removal of the DC part, the reference point of the AC part shown in Figure 2 is centered at zero, and symmetric on the axis of the baseband signal voltage. For illustration, the same reference point shown in Figure 2 can be located at the midpoint between the tag constellation points of State 0 and State 1. Thus, the phase of the received tag signal is defined as follows:

(3) $$\varphi =∡\left({\vec{V}}_{tag}^{1}-{\vec{V}}_{tag}^{0}\right)=\arctan\left(\frac{Q_{ac}}{I_{ac}}\right),\;\;\;\;\varphi \in (-\pi ,\pi ).$$

It has been observed that, when the tag is moved away from or towards the reader, both vectors ${\vec{V}}_{tag}^{1}$ and ${\vec{V}}_{tag}^{0}$ rotate simultaneously and cause change to the phase φ of the received signal. Similarly, φ varies with the relative rotation of the tag antenna linearly, with respect to the reader antenna.

**Figure 4:**
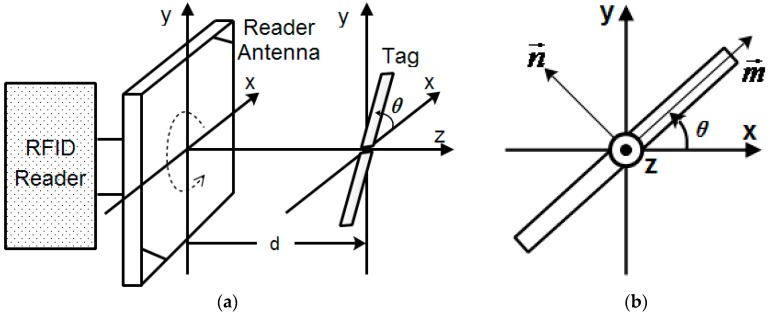
Measurement model: (**a**) Experimental scheme. (**b**) Angular relationship of tag antenna.

In order to study the phase displacement and phase angle characteristics, we propose the measurement model shown in Figure 4. As shown in Figure 4a, the reader antenna is assumed to be a right-hand circular polarization (RHCP) antenna, and the tag antenna is assumed to be a linear polarization antenna. It is assumed that the surface center of the reader antenna is at the origin (0, 0, 0) of the (*x*, *y*, *z*) coordination system. The tag antenna, which is located on the *x*–*y* plane, is parallel to the surface of the reader antenna. Both the reader antenna and the tag antenna are centered and symmetrical on the *z* axis. The distance d between the reader and the tag antenna is measured along the positive *z* axis. In order to estimate the tilt angle of the tag antenna, we consider the vector $\vec{m}$ along the tag antenna in the linear polarization direction, and the vector $\vec{n}$ in the orthogonal direction, as shown in Figure 4b. The angular relationship toward $\vec{m}$ is referred to as the tilt θ, defined by counterclockwise rotation beginning at the positive *x* axis.

### 2.2. Experimental Setup

**Figure 5:**
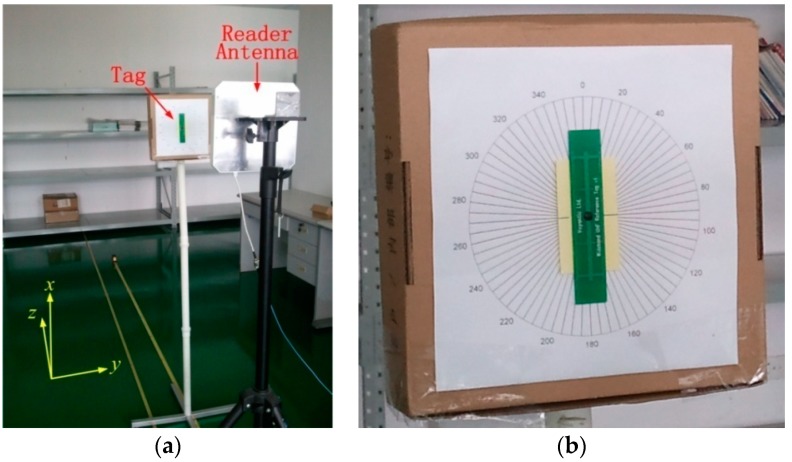
Experimental measurement: (**a**) Test setup. (**b**) Markers illustrating the tag orientation.

In order to verify the calculation mentioned above, we conducted displacement and tilt angle experiments based on the measurement model outlined in Figure 4. All the experiments were carried out in a real-life lab environment with reflecting surfaces (desks, cabinets, and books, etc.), and a photograph of the test scene is shown in Figure 5a. Therefore, the environmental effects, such as multiple paths, noise, and scattering factors, are a realistic part of the scene.

In these experiments, we used a Laird reader antenna [48] tuned to 902–928 MHz (RHCP and 9 dBic gain) and a Voyantic Field Engineer Kit [49] to simulate the process of an RFID system supporting fully coherent detection. As shown in Figure 5a, a reference RFID tag was parallel to the surface of the reader antenna, and accurately controlled for displacement away or towards the reader antenna, at a step size of 2.54 mm. At each step, the reference tag was interrogated by a carrier power of 20 dBm, and replies were sampled continuously by the Voyantic Field Engineer Kit. The same procedure was repeated for a number of tag angular positions at 5° intervals. For illustration purposes, we marked the passive reference tag on a compass of cardboard, as shown in Figure 5b.

According to the propagation characteristics of the RHCP wave in free space, the electric field intensity $\vec{E}$ of the carrier wave, traveling in the positive *z* direction, can be written as [50]

(4) $$\vec{E}=E_{0}e^{j(-\beta z+\varphi_{i})}\left({\vec{a}}_{x}-j{\vec{a}}_{y}\right).$$

Here, $E_{0}$ is the carrier amplitude, and $\varphi_{i}$ is the initial phase. The phase constant is $\beta =\frac{2\pi }{\lambda }$, and λ is the carrier wavelength.

For the tag antenna to have linear polarization in the $\vec{m}$ direction, the received tag signal must have a projection on the $\vec{m}$ direction. That is,

(5) $$\vec{E_{tag}}=AE_{0}e^{j(-\varphi_{d}+\varphi_{i})}(\cos\theta -j\sin\theta ){\vec{a}}_{m}=AE_{0}e^{j(-\varphi_{d}-\theta +\varphi_{i})}{\vec{a}}_{m}.$$

Here, A is the carrier wave propagation attenuation, the signal phase difference is $\varphi_{d}=\frac{2\pi }{\lambda }d$, and d is the distance of the reader to the tag.

According to the characteristics of a linear polarization antenna, the electric field intensity $\vec{E_{bc}}$ of the arriving wave at the surface of the reader antenna, backscattered by the linearly polarized tag antenna in the $\vec{m}$ direction, can be written as

(6) $$\vec{E_{bc}}=A^{\prime }AE_{0}S_{t}e^{j(-2\varphi_{d}-\theta +\varphi_{i}-\varphi_{bc})}{\vec{a}}_{m}.$$

Here, $A^{\prime }$ is the attenuation of the backscattered path, and $\varphi_{bc}$ is the phase delay during the backscattering process of the tag. $S_{t}$ is the reflection coefficient of the tag response, which is decided by tag states. If the tag is in matching state (State 0), then $S_{t}=S_{0}$. Otherwise, when the tag is in mismatching state (State 1), $S_{t}=S_{1}$. It is assumed that the total reader-to-tag-to-reader path is a monostatic channel and has no multipath effect. Thus, the reader-to-tag and tag-to-reader distances can be assumed to be d. Hence, the phase difference of the total propagation path is $2\varphi_{d}$.

The electric field intensity $\vec{E_{bc}}$ of the linearly polarized arrival wave can also be represented as $\vec{E_{bc}}=\vec{E_{bcL}}+\vec{E_{bcR}}$. $\vec{E_{bcL}}$ and $\vec{E_{bcR}}$ are, respectively, the left-hand circular polarization (LHCP) and RCHP wave components in the negative *z* direction. According to Equation (6), they can be written as

(7) $$\left\{ \begin{array}{l} \vec{E_{bcL}}=\frac{1}{2}A^{\prime }AE_{0}S_{t}e^{j(-2\varphi_{d}-\theta +\varphi_{i}-\varphi_{bc})}({\vec{a}}_{m}-j{\vec{a}}_{n})\\ \vec{E_{bcR}}=\frac{1}{2}A^{\prime }AE_{0}S_{t}e^{j(-2\varphi_{d}-\theta +\varphi_{i}-\varphi_{bc})}({\vec{a}}_{m}+j{\vec{a}}_{n}). \end{array}\right.$$

The two orthogonal vectors, ${\vec{a}}_{m}$ and ${\vec{a}}_{n}$, are related to the *x–y* coordinate system by

(8) $$\left\{ \begin{array}{l} {\vec{a}}_{m}={\vec{a}}_{x}\cos\theta +{\vec{a}}_{y}\sin\theta \\ {\vec{a}}_{n}=-{\vec{a}}_{x}\sin\theta +{\vec{a}}_{y}\cos\theta_{}. \end{array}\right.$$

Since the reader antenna is assumed to be an RHCP antenna and only receive the RCHP component of the arriving wave, we only consider $\vec{E_{bcR}}$. For coordinates transformed by Equation (8), this can be expressed as

(9) $$\vec{E_{bcR}}=\frac{1}{2}A^{\prime }AE_{0}S_{t}e^{j(-2\varphi_{d}-2\theta +\varphi_{i}-\varphi_{bc})}({\vec{a}}_{x}+j{\vec{a}}_{y}).$$

According to Equation (9), the instantaneous scalar expression of the received signal arriving at the reader can be illustrated as [42]

(10) $$y(t)=\frac{1}{2}A^{\prime }AE_{0}S_{t}\cos(\omega_{}t-2\varphi_{d}-2\theta +\varphi_{i}-\varphi_{bc}).$$

Meanwhile, considering Equation (3), the in-phase and quadrature component of the received signal can also be written as

(11) $$\left\{ \begin{array}{l} I=\frac{1}{4}A^{\prime }AE_{0}S\cos\varphi \\ Q=\frac{1}{4}A^{\prime }AE_{0}S\sin\varphi_{}. \end{array}\right.$$

Considering $\varphi_{d}=\frac{2\pi }{\lambda }d(t)$, we compare Equation (10) with Equation (11), and draw a conclusion that the received tag signal phase φ(t) is related to the reader-to-tag distance d(t) and the tag tilt angle θ(t) by

(12) $$\varphi (t)=\frac{4\pi }{\lambda }d(t)+2\theta (t)-(\varphi_{i}-\varphi_{bc}-\varphi_{o}).$$

Here, $\varphi_{o}$ is a constant phase offset of the demodulation process, and φ(t) has an unknown integer number of 2π radian offsets, due to the cycle ambiguity.

## 3. Results

### 3.1. Displacement Experiment

We first investigated the received tag signal phase φ(t) as a function of displacement. The original distance from tag to reader was fixed at $\hat{d}(0)=50\;\mathrm{cm}$, and displacement was defined as $\Delta \hat{d}=\hat{d}(t)-\hat{d}(0)$, $\Delta \hat{d}\in (-\frac{\lambda }{4},\frac{\lambda }{4}]$. Meanwhile, the tilt angle was defined as $\Delta \hat{\theta }=\hat{\theta }(t)-\hat{\theta }(0)$, $\Delta \hat{\theta }\in (-\frac{\pi }{2},\frac{\pi }{2}]$. For consistent estimation, the tag tilt was fixed to $\hat{\theta }(t)\equiv 90^{{}^{\circ}}$, which leads to $\Delta \hat{\theta }=0$. It is clear from Equation (3) that the estimated phase $\hat{\varphi }(t)$ is unambiguous, and that $\hat{\varphi }(t)\in (-\pi ,\pi ]$. Considering $\Delta \hat{d}\in (-\frac{\lambda }{4},\frac{\lambda }{4}]$ and $\hat{\varphi }(t)\in (-\pi ,\pi ]$, the estimation range is given by

(13) $$-2\pi <\frac{4\pi }{\lambda }\Delta \hat{d}+\hat{\varphi }(0)\leq 2\pi .$$

According to Equations (12) and (13), the estimated phase $\hat{\varphi }(t)$ can be expressed by

(14) $$\hat{\varphi }(t)=\left\{ \begin{array}{l} \frac{4\pi }{\lambda }\Delta \hat{d}+\hat{\varphi }(0)+2\pi ，\left[\frac{4\pi }{\lambda }\Delta \hat{d}+\hat{\varphi }(0)\right]\in (-2\pi ,-\pi ]\\ \frac{4\pi }{\lambda }\Delta \hat{d}+\hat{\varphi }(0)，\left[\frac{4\pi }{\lambda }\Delta \hat{d}+\hat{\varphi }(0)\right]\in (-\pi ,\pi ]\\ \frac{4\pi }{\lambda }\Delta \hat{d}+\hat{\varphi }(0)-2\pi ，\left[\frac{4\pi }{\lambda }\Delta \hat{d}+\hat{\varphi }(0)\right]\in (\pi ,2\pi ]. \end{array}\right.$$

Here, the carrier wavelength λ can be seen as constant, and the estimated phase $\hat{\varphi }(t)$ is then proportional to $\Delta \hat{d}$.

**Figure 6:**
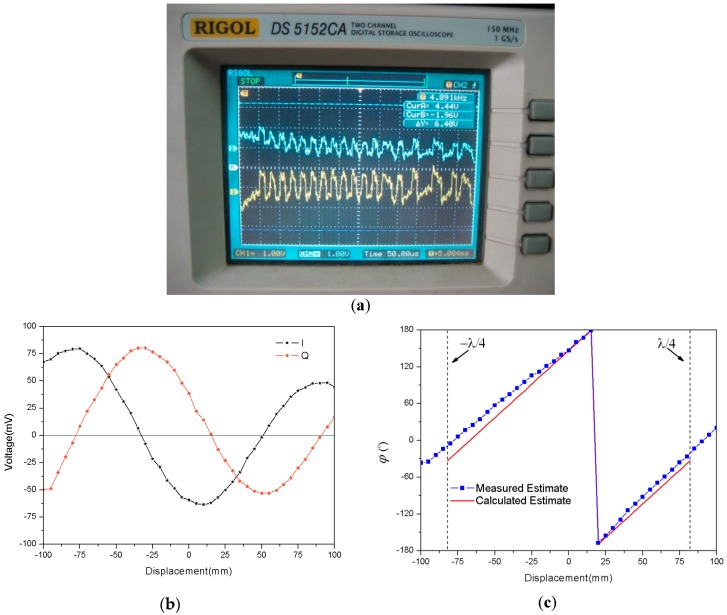
Displacement measured results: (**a**) Original source of measurement data; (**b**) Sampled voltages at I/Q channel; (**c**) Estimated phase φ as a function of displacement.

As shown in Figure 6a, the sampled peak–peak voltages at the I/Q channel are given at each step of displacement. We found that sampled curves of the I channel and Q channel are both sine wave curves, and are orthogonal to each other. Moreover, the maximum amplitude of the sine wave curves attenuates quickly with the increase of Δd.

**Figure 7:**
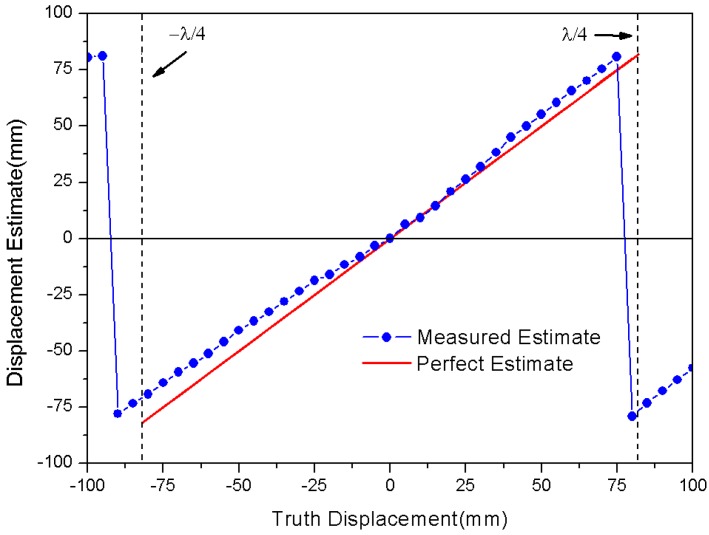
Comparison between measured and perfect estimated displacement.

With the measurement method of Figure 6a, original data can be obtained for the displacement measurement, which leads to Figure 6b,c. Based on these sampled voltages in Figure 6b, we can obtain the measured estimate $\hat{\varphi }(t)$ by Equation (3), as shown in Figure 6c. Meanwhile, according to Equation (14), we can calculate the estimate $\hat{\varphi }(t)$ as a function of truth displacement Δd, plotted in Figure 6c. There is a reasonable agreement between the measured and calculated estimates for the received signal phase $\hat{\varphi }(t)$. The measured estimate results seem to follow a linear trend, and have estimation errors at both ends of the truth displacement Δd due to the phase error accumulation effect. These results primarily validate the correctness of Equation (14), and we can obtain the measured estimate $\Delta \hat{d}$ from the measured estimate $\hat{\varphi }(t)$ by using Equation (14). As shown in Figure 7, the measured estimate $\Delta \hat{d}$ appears to be fairly linear, and approaches truth displacement Δd. Therefore, it is apparent that the phase parameter of the RFID received signal can be measured for detection of displacement variation, which makes an RFID tag into a displacement sensor.

### 3.2. Tilt Angle Experiment

In the following, we consider the received tag signal φ(t) as a function of tilt angle. The definitions of displacement and tilt angle are the same as in the previous measurement. For consistent estimation, we fixed the reader-to-tag distance at $\hat{d}(t)\equiv 0.5\;m$, and then $\Delta \hat{d}=0$. Considering $\Delta \hat{\theta }\in (-\frac{\pi }{2},\frac{\pi }{2}]$ and $\hat{\varphi }(t)\in (-\pi ,\pi ]$, the estimation range is given by

(15) $$-2\pi <2\Delta \hat{\theta }+\hat{\varphi }(0)\leq 2\pi .$$

From Equation (12) and Equation (15), the estimated phase $\hat{\varphi }(t)$ can be expressed by

(16) $$\hat{\varphi }(t)=\left\{ \begin{array}{l} 2\Delta \hat{\theta }+\hat{\varphi }(0)+2\pi ，\left[2\Delta \hat{\theta }+\hat{\varphi }(0)\right]\in (-2\pi ,-\pi ]\\ 2\Delta \hat{\theta }+\hat{\varphi }(0)，\left[2\Delta \hat{\theta }+\hat{\varphi }(0)\right]\in (-\pi ,\pi ]\\ 2\Delta \hat{\theta }+\hat{\varphi }(0)-2\pi ，\left[2\Delta \hat{\theta }+\hat{\varphi }(0)\right]\in (\pi ,2\pi ]. \end{array}\right.$$

Here, the carrier wavelength λ can be seen as constant, and the estimated phase $\hat{\varphi }(t)$ is then proportional to the tilt angle $\Delta \hat{\theta }$.

**Figure 8:**
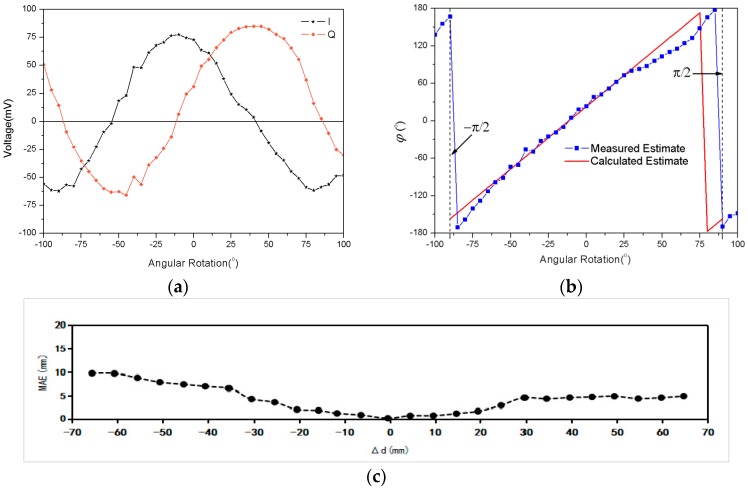
Tilt angle results: (**a**) Sampled voltages at the I/Q channel; (**b**) Estimated phase φ as a function of tilt angle; (**c**) Mean absolute error (MAE) result between measured and calculated estimate.

**Figure 9:**
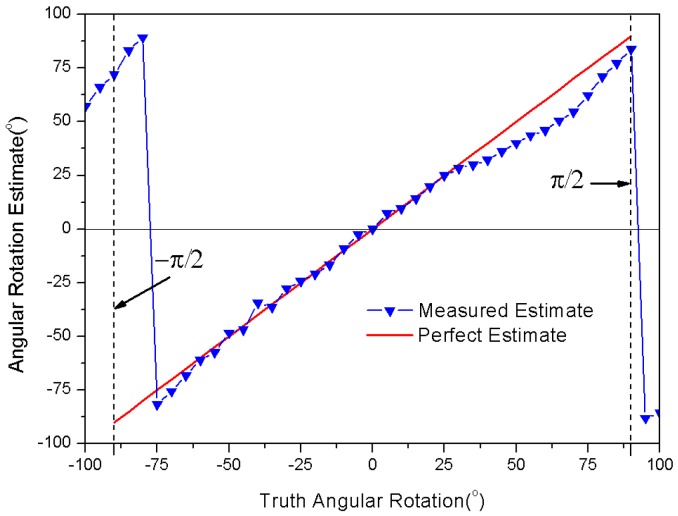
Comparison between measured and perfect estimated tilt angle.

As shown in Figure 8a, the sampled peak–peak voltages at the I/Q channel are given at each interval of the tilt angle, and the reader-to-tag distance maintains constant at $\hat{d}(t)\equiv 50\;\mathrm{cm}$. Based on these sampled voltages in Figure 8a, we can obtain the measured estimate $\hat{\varphi }(t)$ by Equation (3), as shown in Figure 8b. Meanwhile, according to Equation (16), we can calculate the estimate $\hat{\varphi }(t)$ as a function of the truth tilt angle Δθ, plotted in Figure 8b. There is a reasonable agreement between the measured and calculated estimate for the received signal phase $\hat{\varphi }(t)$, except between the positions Δθ=π/4 and Δθ=π/2. The significant fluctuations occur due to the polarization mismatch of the reader antenna. These results primarily validate the correctness of Equation (16), and we can obtain the measured estimate $\Delta \hat{\theta }$ from the measured estimate $\hat{\varphi }(t)$ by using Equation (16). As shown in Figure 9, the measured estimate $\Delta \hat{\theta }$ appears to be quite linear, and agrees well with the truth tilt angle Δθ, except between the positions Δθ=π/4 and Δθ=π/2. If we consider the antenna polarization mismatch effect, the RFID received signal phase can be seen as robust for the detection of angular variation, and can serve as an angular sensor.

## 4. Discussion

**Figure 10:**
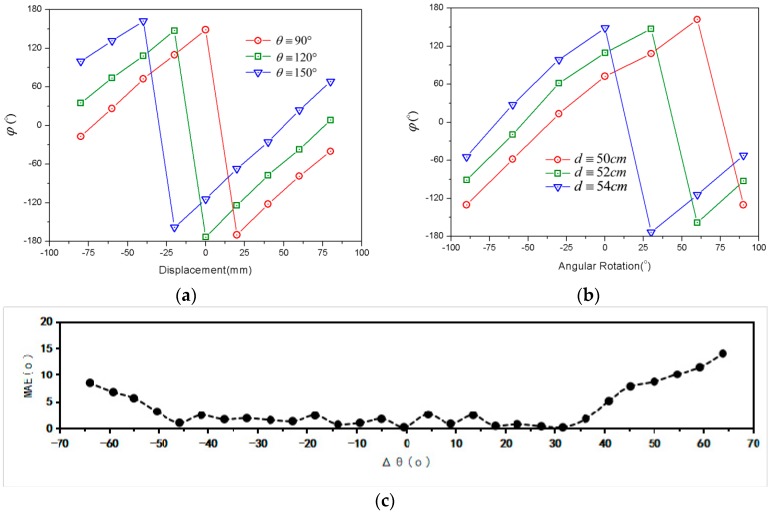
Experimental results with different limitation conditions: (**a**) Displacement curves with different tilt limitations; (**b**) Angular curves with different position limitations; (**c**) MAE result between measured and calculated estimate for Δθ.

In previous experiments, measurement results of displacement were tested with a fixed tilt angle, and tilt angle data were tested with a static distance. It is necessary to consider the proposed method with different limitation conditions. Experimental results are illustrated in Figure 10. With the increments of initial tilt and distance, the displacement and tilt angle curves are shifted to the left. It should be noted that due to the variation of the limitation condition, the received tag signal phase will not remain unique at a certain position, as shown in Figure 10. It is very difficult to separate the angular effect from displacement effects. Thus, the location results by using the phase-based RFID technique [37,38] are not robust, since they did not consider the effect of tag antenna polarization direction. For consistent purposes, it is desirable that the displacement sensor works with fixed tilt, and the angular sensor with static distance. As shown in Figure 10c, assume that the variation of the tilt angle is less than 30°; then, the MAE of the tilt angle is less than 2.5° for an RFID system with 500 mm working range.

As shown in Table 1, the sensing capability and measurement precision of this paper were compared with previous works. With different system schemes and different measurement methods, a passive RFID system can be used in different sensing applications.

## 5. Conclusions

In this paper, a novel method for displacement and tilt detection has been validated through theoretic analysis and experiment. A conventional RFID tag attached to an object and an UHF RFID reader were used to compose the proposed detection system. The displacement and tilt angle were estimated respectively using LP and CP reader antennas. With a linear polarization reader antenna, the relation between the phase parameter of the received RFID tag signal and displacement can be proved to be approximately linear through experimental results. Based on previous displacement results, the phase–angle relation of the tag’s backscatter wave, which is close to linear, can also be obtained by a circular polarization reader antenna. Thus, the proposed method can obtain not only the displacement, but also the tilt angle of the tag. It is a reliable and low-cost solution for wireless monitoring applications.

## Figures and Tables

**Table 1:** Comparison of sensing capability and measurement precision.

Measurement Methods	Displacement Sensing	Tilt Angle Sensing	Measurement Precision of Displacement	Measurement Precision of Tilt Angle
Double phase difference [36]	Yes	NA	6° (phase) ^1^	NA
Transmitted power monitoring [37]	Yes	NA	2% (Power) ^1^	NA
Received signal strength [43]	NA	Yes	NA	7°
Antenna polarization [44]	NA	Yes	NA	15°
[this paper]	Yes	Yes	2 mm	2.5°

^1^ Conversion is required for displacement data.

## References

[1] Komninos N. (2003). Intelligent Cities.

[2] Atzori L., Iera A., Morabito G. (2010). The internet of things: A survey. Comput. Netw..

[3] Musa A., Gunasekaran A., Yusuf Y. (2014). Supply chain product visibility: Methods, systems and impacts. Expert Syst. Appl..

[4] Chang P.C., Flatau A., Liu S.C. (2003). Review paper: Health monitoring of civil infrastructure. Struct. Health Monit..

[5] Bhuiyan M.Z.A., Wang G., Cao J. (2015). Deploying wireless sensor networks with fault-tolerance for structural health monitoring. IEEE Trans. Comput..

[6] Evers L., Havinga P.J.M., Kuper J. SensorScheme: Supply chain management automation using wireless sensor networks. Proceedings of the IEEE International Conference on Emerging Technologies and Factory Automation.

[7] Finkenzeller K., Waddington R. (2004). RFID Handbook: Radio-Frequency Identification Fundamentals and Applications.

[8] Kim S., Mariotti C., Alimenti F., Mezzanotte P., Georgiadis A., Collado A., Roselli L., Tentzeris M.M. (2013). No battery required: Perpetual RFID-enabled wireless sensors for cognitive intelligence applications. IEEE Microw. Mag..

[9] Occhiuzzi C., Caizzone S., Marrocco G. (2013). Passive UHF RFID antennas for sensing applications: Principles, methods and classifications. IEEE Antennas Propag. Mag..

[10] Chang K., Kim Y.-H., Kim Y.-J., Yoon Y.J. (2007). Functional antenna integrated with relative humidity sensor using synthesised polyimide for passive RFID sensing. Electron. Lett..

[11] Manzari S., Occhiuzzi C., Nawale S., Catini A., Di Natale C., Marrocco G. Polymer-doped UHF RFID tag for wireless-sensing of humidity. Proceedings of the IEEE International Conference on RFID (RFID).

[12] Sauer S., Fischer W.J. (2016). An irreversible single-use humidity-threshold monitoring sensor principle for wireless passive sensor solutions. IEEE Sens. J..

[13] Siddiqui A., Mahboob R., Islam T. (2017). A passive wireless tag with digital readout unit for wide range humidity measurement. IEEE Trans. Instrum. Meas..

[14] Bhattacharyya R., Floerkemeier C., Sarma S., Deavours D. RFID tag antenna based temperature sensing. Proceedings of the IEEE International Conference on RFID (RFID).

[15] Babar A.A., Manzari S., Sydanheimo L., Elsherbeni A.Z., Ukkonen L. (2012). Passive UHF RFID tag for heat sensing applications. IEEE Trans. Antennas Propag..

[16] Vena A., Sydänheimo L., Tentzeris M.M., Ukkonen L. (2015). A fully inkjet-printed wireless and chipless sensor for CO_2_ and temperature detection. IEEE Sens. J..

[17] Abad E., Zampolli S., Marco S., Scorzoni A., Mazzolai B., Juarros A., Gómez D., Elmi I., Cardinali G.C., Gómez J.M. (2007). Flexible tag micro lab development: Gas sensors integration in RFID flexible tags for food logistic. Sens. Actuators B.

[18] Occhiuzzi C., Rida A., Marrocco G., Tentzeris M. (2011). RFID passive gas sensor integrating carbon nanotubes. IEEE Trans. Microw. Theory Tech..

[19] Occhiuzzi C., Paggi C., Marrocco G. (2011). Passive RFID strain-sensor based on meander-line antennas. IEEE Trans. Antennas Propag..

[20] Occhiuzzi C., Marrocco G. (2013). Constrained-design of passive UHF RFID sensor antennas. IEEE Trans. Antennas Propag..

[21] Chin J.-C., Rautenberg J.M., Ma C.Y., Pujol S., Yau D.K. (2009). An experimental low-cost, low-data-rate rapid structural assessment network. IEEE Sens. J..

[22] Suwalak R., Phongcharoenpanich C., Torrungrueng D., Krairiksh M. (2012). Determination of dielectric property of construction material products using a novel RFID sensor. Prog. Electromagn. Res..

[23] Cook B.S., Cooper J.R., Tentzeris M.M. (2013). An inkjet-printed microfluidic RFID-enabled platform for wireless lab-on-chip applications. IEEE Trans. Microw. Theory Tech..

[24] Occhiuzzi C., Marrocco G. (2016). Precision and accuracy in UHF-RFID power measurements for passive sensing. IEEE Sens. J..

[25] Okada M. Mitigation of detection error in RFID-based in-body localization system. Proceedings of the 9th Biomedical Engineering International Conference (BMEiCON).

[26] Kalansuriya P., Bhattacharyya R., Sarma S. (2013). RFID tag antenna-based sensing for pervasive surface crack detection. IEEE Sens. J..

[27] Paolini G., del Prete M., Berra F., Masotti D., Costanzo A. An agile and accurate microwave system for tracking elderly people occupancy at home. Proceedings of the IEEE MTT-S Latin America Microwave Conference (LAMC).

[28] Caizzone S., DiGiampaolo E., Marrocco G. (2014). Wireless crack monitoring by stationary phase measurements from coupled RFID tags. IEEE Trans. Antennas Propag..

[29] Wang C., Xie L., Wang W., Xue T., Lu S. Moving tag detection via physical layer analysis for large-scale RFID systems. Proceedings of the IEEE International Conference on Computer Communications.

[30] Wegener M., Froß D., Rößler M., Drechsler C., Pätz C., Heinkel U. Relative localisation of passive UHF-tags by phase tracking. Proceedings of the International Multi-Conference on Systems, Signals & Devices (SSD).

[31] Genovesi S., Costa F., Borgese M., Monorchio A., Manara M. Chipless RFID tag exploiting cross polarization for angular rotation sensing. Proceedings of the 2016 IEEE International Conference on Wireless for Space and Extreme Environments (WiSEE).

[32] Ahmed R., Avaritsiotis J.N. The propagation parameters on RFID-localization accuracy. Proceedings of the Science and Information Conference (SAI).

[33] Khanam S., Mahbub M., Mandal A., Kaiser M.S., Mamun S.A. Improvement of RFID tag detection using smart antenna for tag based school monitoring system. Proceedings of the International Conference on Electrical Engineering and Information & Communication Technology.

[34] Rizzoli V., Costanzo A., Montanari E., Benedetti A. (2009). A new wireless displacement sensor based on reverse design of microwave and millimeter-wave antenna array. IEEE Sens. J..

[35] Han J., Qian C., Wang X., Ma D., Zhao J., Zhang P., Xi W., Jiang Z. Twins: Device-free object tracking using passive tags. Proceedings of the IEEE/ACM Transactions on Networking.

[36] Caizzone S., Giampaolo E.D., Marrocco G. (2017). Setup-independent phase-based sensing by UHF RFID. IEEE Antennas Wirel. Propag. Lett..

[37] Cazeca M.J., Mead J., Chen J., Nagarajan R. (2013). Passive wireless displacement sensor based on RFID technology. Sens. Actuators A.

[38] Cook B.S., Shamim A., Tentzeris M.M. (2012). Passive low-cost inkjet-printed smart skin sensor for structural health monitoring. IET Microw. Antennas Propag..

[39] Becker J., Trotter M.S., Griffin J.D. Passive displacement sensing using backscatter RFID with multiple loads. Proceedings of the IEEE SENSORS.

[40] Paggi C., Occhiuzzi C., Marrocco G. (2014). Sub-millimeter displacement sensing by passive UHF RFID antennas. IEEE Trans. Antennas Propag..

[41] Perret E. (2017). Displacement sensor based on radar cross-polarization measurements. IEEE Trans. Microw. Theory Tech..

[42] UHF Frequency Regulations—GS1. http://www.gs1.org/docs/epcglobal/UHF_Regulations.pdf.

[43] Krigslund R., Popovski P., Pedersen G.F. (2012). Orientation sensing using multiple passive rfid tags. IEEE Antennas Wirel. Propag. Lett..

[44] Gupta G., Singh B.P., Bal A. (2014). Orientation detection using passive UHF RFID technology. IEEE Aantennas Propag. Mag..

[45] Krigslund R., Dosen S., Popovski P. (2013). A novel technology for motion capture using passive UHF RFID tags. IEEE Trans. Biomed. Eng..

[46] Li X., Zhang Y., Amin M.G. Multi frequency-based range estimation of RFID tags. Proceedings of the IEEE International Conference on RFID (RFID).

[47] Nikitin P.V., Martinez R., Ramamurthy S., Leland H., Spiess G., Rao K. Phase based spatial identification of UHF RFID tags. Proceedings of the IEEE International Conference on RFID (RFID).

[48] Circular Polarity RFID Panel Antenna S9028PCL S9028PCR. https://assets.lairdtech.com/home/brandworld/files/ANT-DS-S9028PCL%20S9028PCR-0515.pdf.

[49] Field Engineer Kit. http://voyantic.com/products/tagformance-pro/accessories/field-engineer-kit.

[50] Balanis C.A. (2005). Antenna Theory: Analysis and Design.

